# Trajectory Shifts in Interdisciplinary Research of the Aryl Hydrocarbon Receptor—A Personal Perspective on Thymus and Skin

**DOI:** 10.3390/ijms22041844

**Published:** 2021-02-12

**Authors:** Charlotte Esser

**Affiliations:** IUF-Leibniz Research Institute for Environmental Medicine, Auf’m Hennekamp 50, 40225 Düsseldorf, Germany; Charlotte.Esser@iuf-duesseldorf.de; Tel.: +49-211-3389-253

**Keywords:** thymus, immune system, skin, 2,3,7,8-tetrachlorodibenzo-*p*-dioxin (TCDD), aryl hydrocarbon receptor (AHR), therapeutic target

## Abstract

Identifying historical trajectories is a useful exercise in research, as it helps clarify important, perhaps even “paradigmatic”, shifts in thinking and moving forward in science. In this review, the development of research regarding the role of the transcription factor “aryl hydrocarbon receptor” (AHR) as a mediator of the toxicity of environmental pollution towards a link between the environment and a healthy adaptive response of the immune system and the skin is discussed. From this fascinating development, the opportunities for targeting the AHR in the therapy of many diseases become clear.

## 1. Introduction

Identifying historical trajectories is a useful exercise in research, as it helps clarify important, perhaps even “paradigmatic shifts” in thinking and moving forward in science. Here, I discuss this for the aryl hydrocarbon receptor (AHR), an evolutionary ancient transcription factor. AHR research underwent a fascinating development, moving from (i) a protein of interest for toxicologists studying the health impact of environmental pollutants, i.e., polycyclic aromatic hydrocarbons (PAHs), to (ii) a transcription factor recognized as a player in immunology and many more physiological functions, to (iii) a molecular target for a broad range of diseases. I will here focus on AHR and immunotoxicology/immunology, exemplified by the thymus and skin, in a reflection of my own research over three decades.

## 2. 2,3,7,8-tetrachlorodibenzo-*p*-dioxn (TCDD) and the Thymus 1990–2000

I entered the field of toxicology in the early 1990s, with a strong basic immunology background. Environmental levels of the anthropogenic pollutant 2,3,7,8-tetrachlorodibenzo-*p*-dioxin (TCDD) and other dioxins, furans, or polyhalogenated biphenyls and PAHs were unacceptably high in industrialized countries. Some even called TCDD the “super-poison” since the explosion accident in Meda, Italy in 1976 [1], which brought the very high toxicity of TCDD to the attention of the general public. After this explosion at the small chemical plant *Industrie Chimiche Meda Società Azionaria* (ICMESA) in Meda, near Milan, Italy, approximately 1 kg of TCDD killed animals in the surrounding area (municipality of Seveso) of several square kilometers, and led to chloracne in humans. Long-term effects over the years resulted in many adverse health effects, including cancer and reproductive issues [2]. The Seveso incident led to novel European regulations, known as the Seveso III directive (see https://eur-lex.europa.eu/legal-content/EN/TXT/?uri=celex%3A32012L0018 (last accessed on 10 February 2021)). There had been previous incidents and accidents with TCDD and “dioxin-like” substances, and others were to follow, such as the contamination of poultry feed in Belgium in 1999 [3]. For a list, see Table 1 in the 2020 review by Chris Bradfield´s group [4]. In Germany, public funding strategies for dioxins, furans and biphenyls mainly supported the analysis, avoidance of and reduction in emissions, as well as research on cancer, reproductive health, and immunotoxicology. Skin was not a focus, maybe because chloracne is not an issue in low-dose environmental exposure to the general public. It was well-established in the early 1990s that TCDD causes thymus atrophy and immunosuppression [5,6]. Both are hallmarks of dioxin exposure, found in many animals, even at low levels. The existence of an intracellular receptor for TCDD—called the “dioxin receptor”, now the “AHR” —was known, as well as mouse strains with different sensitivities to TCDD and correlated AHR-polymorphisms *Ahr^d^*, *Ahr^b^*^-1^, and *Ahr^b^*^-2^ [7,8]. Whether there was a link between thymus atrophy and the TCDD-mediated immunosuppression was unclear, and indeed, the underlying molecular causes of acute high-dose dioxin toxicity—cachexia, even death (in animals), or chloracne (in humans)—were an enigma. Using my immunological fascination with immune cell development and their potential functional consequences, my first papers were on the shifts in the dynamics of thymocyte development. I identified CD4/CD8 subsets in the thymus by flow cytometry upon dioxin receptor induction, and how basic immunological phenomena like positive and negative selection of thymocytes might be changed [9,10]. This seemed to be the path towards a grasp of the overall immunosuppressive “effect” of dioxin. In addition, I used TCDD/AHR as a tool to understand the role of AHR for an important immunological process, thymocyte developmental pathways and, thus, the generation of immunocompetent T cells. I was not alone in this. Seminal work by the groups of William Greenlee or Allen Silverstone (USA), and Lennart Dencker or Stan Orrenius (Sweden) had studied the apoptotic and developmental effects of TCDD on the thymocytes [11,12,13]. The expression of AHR (at the time still called “a specific binding protein for TCDD”) in thymic epithelial cells was shown, as well as the direct action of TCDD on these cells, which resulted in impaired maturation of thymocytes [14,15,16]. TCDD and thymocyte development remained a topic of intense research for years to come. The focuses were the impairment of thymocyte emigration [17], of cell cycle arrest [18], enhancement of selection [13,19] or the requirement of the c-src pathway [20]. Other basic subjects, like the discovery of AHR nuclear translocator (ARNT) [21], or highly differential tissue expression of AHR—with high expression in the thymus—also came to light in the 1990s [22]. Moreover, beautiful work from several groups worked out the basic signaling pathway of AHR in liver cells—binding to the ligand, translocation to the nucleus, dimerization, binding to DNA, transcription initiation, and induction of a battery of xenobiotic metabolizing enzymes, and addressed questions such as competition between AHR, ARNT and other dimerization partners [23]. Table 1 summarizes major milestones in AHR biochemistry and gives more references. In immunotoxicology, an important question was whether the impaired development and output of T cells from the thymus leads to immunosuppression or autoimmunity [24,25]. This remains unsolved, although a single cause–effect relationship seems unlikely, given the many dampening effects TCDD has on other immune cells. Indeed, at a first meeting dedicated solely to dioxin and the immune response, which took place at my institute in Düsseldorf, Germany, in 1994, such questions were central [6]. An answer would also have been consequential in regulatory or political terms. For instance, the herbicide Agent Orange was sprayed from 1962 to 1971 during the Vietnam War by the U.S. forces to lay waste to land and make the opposing Viet Cong more vulnerable to military operations [26]. Agent Orange contains TCDD as a contaminant, and soon after the war, U.S. veterans wanted to know about their health and establish lines of responsibility [27]. However, even decades later, the report of the U.S. American committee charged with reviewing the data in 2018 stated that evidence to determine an association between autoimmunity and this exposure of military personnel “remains inadequate or insufficient” [28]. 

There are a number of difficulties, which hinder assessing the “immunotoxicity” of environmental chemicals in humans. These are the exact exposure details (often unknown), limited epidemiological data, or simply a lack of tools or knowledge to quantify or determine the right immunological parameters. Measuring antibody titers or frequency of T cells or Natural Killer cells in the blood, and similar stand-ins for “immune function” are not necessarily meaningful and, indeed, it is hard to define a clinical threshold for such parameters [56]. In experimental animal infection studies, research looked at the impaired systemic immune response towards bacterial and viral [57], and found such impairments. This also helped explain immunosuppression in wildlife fish or mammals, such as Baltic seals, *Phoca vitulina,* feeding on highly contaminated herring [58]. Nonetheless, although we know without doubt from many laboratory animals, including even the primate species *Callythrix jacchus* (marmosets) [59], that TCDD and dioxin-like chemicals are immunosuppressive at low doses, it remains a challenge to quantify this dimension for humans. Curiously, TCDD (but not plant-derived ligands such as indoles) binds the human AHR with a comparatively lower affinity than in many animals. In 2016, Gary Perdew and colleagues, based on genetic comparisons of the extinct *Homo neandertaliensis* and the extant *Homo sapiens,* suggested that this might have been an evolutionary advantage gained after humans began to use fire and regularly breathed its halogenated polycyclic aromatic hydrocarbons (HPAH) and PAH-containing toxic smoke [52]. The lethal acute dose of TCDD poisoning in humans is unknown. The highest dose recorded in a person so far is 144,000 pg/g blood fat, i.e., 25 μg/kg body weight [60]. In 2001, the WHO established a provisional tolerable monthly intake (PTMI) of 70 picogram/kg per month for humans (see https://www.who.int/news-room/fact-sheets/detail/dioxins-and-their-effects-on-human-health (last accessed on 11 Februrary 2021)). 

## 3. A Shift from Toxicology towards Immunology—Aryl Hydrocarbon Receptor (AHR) Research in the New Century

Despite the research focus on TCDD and other dioxin-like compounds, it appeared self-evident that AHR functions must go far beyond being a “dioxin receptor” and initiator of detoxifying anthropogenic compounds. This was due simply to the fact that AHR is, evolutionarily, very old, and although forest fires can generate dioxins and PAHs, this would not have generated enough selection pressure to explain such a widespread molecule being present across the animal kingdom. AHR molecules are present not only in vertebrates, but also in nematodes and insects, even in fungi [61]. Regarding the immune system in vertebrates, TCDD research had also firmly established the concept of “cell specificity”, i.e., that it matters for the health outcome of dioxin exposure whether the receptor is present at all, and in which, cells and tissues. AHR expression levels are high in the liver, i.e., the organ for metabolization and detoxification. However, differential expression analysis also demonstrated high AHR expression in the thymus, the lung, the gut and the skin, even the placenta [45,62]. Of note, these are barrier organs interacting with the environment, suggestive of function(s) in environmental adaption. In any case, in the middle of the first decade of the new century, strictly TCDD-focussed immunotoxicological research decreased, and research on the physiology of AHR, including for the immune system, began to rise and took center stage. Three papers in the high-ranking journals *Nature* and *Proc. Natl. Acad. Sci*, independently published in 2008 by the groups of Francisco Quintana and Howard Weiner in the U.S.A., Gitta Stockinger in England, and Tadamitsu Kishimoto in Japan, were the rapid catalysators of this change, which had slowly begun before [63,64,65]. Now, AHR became of interest for the “basic” immunology community, and AHR research almost exploded. These groups described the seminal discovery of the AHR-mediated differentiation of T helper (Th)17 cells, a subset of T cells themselves that was identified only around 2005 [66]. The discovery of Th17 cells first linked them to autoimmune diseases, albeit Th17´s real function is host defense against infections. AHR is well expressed in Th17 cells (but not in Th1 or Th2 cells) [64], and both in vitro activation studies and studies with AHR-deficient cells demonstrated its critical role for the production of the pro-inflammatory cytokine interleukin (IL)-22 in an experimental autoimmune encephalitis (EAE) disease setting. Moreover, treatment of mice with TCDD ameliorated EAE by the induction of regulatory T cells (Treg), yet treatment with formylindolo[2,3-b]carbazol (FICZ), an endogenous high-affinity AHR ligand, boosted Th17 differentiation [63]. In AHR-deficient mice, EAE pathology, compared to wildtype mice, was lower. Immediately, both the Quintana and Stockinger groups identified AHR as a therapeutic target for immunomodulation, a dramatic shift from the previous perception of AHR-activation (by TCDD) as deeply toxic and a major health threat. However, it remained a puzzle why two high-affinity AHR-agonists–TCDD and FICZ–led to juxtaposed outcomes in EAE. Later work on ligands showed that the duration and persistence of ligand-binding, rather than mere affinity, is relevant, as well as parallel effects on several immune cell types, i.e., T cells and dendritic cells in vivo [67,68]. 

### Therapeutic Opportunities on the Horizon

Somewhat earlier, Nancy Kerkvliet and her group had already studied the effects of TCDD on the formation of Treg cells. For instance, they reported that the adoptive transfer of T cells into TCDD-treated mice is accompanied by the transient generation of a population of CD4+CD25+ cells with regulatory properties [69]. Other studies demonstrated that AHR induces Tr1 cells by acting in synergy with c-maf [70,71]. Tr1 cells are CD4+FoxP3-negative Treg cells, induced in the periphery, and they AHR-dependently secrete large amounts of IL-10 [70,72]. In the wake of the work on Th17 and Treg cells, many papers elucidated the pivotal role of AHR signaling on the immune system, in particular for T cells, B cells, and innate immune cells, especially dendritic cells (DC) (for details, see reviews [73,74,75]). DC takes up antigen, an event that triggers their maturation. They then present the antigen to T cells, while secreting cytokines, which drive T cell differentiation towards Th1, Th2, Th17 or Treg. AHR activation inhibits DC´s in vitro differentiation [76]. Experiments with human monocyte-derived DC showed that a small molecular-weight drug and AHR ligand, VAF347 ((4-(3-Chloro-phenyl)-pyrimidin-2-yl)-(4-trifluoromethyl-phenyl)-amine))**,** could block IL-6 secretion, CD86 and HLA-DR upregulation, resulting in impaired T cell stimulation by the DC [76]. In such AHR-inhibitor studies, the therapeutic potential of AHR was the focus, and the search for small molecules expanded, which are AHR-activators, inhibitors or selective modulators. Discoveries also identified unexpected physiological ligands. Mouse DC produces the enzyme 2,3-indoleaminedioxygenase (IDO) in an AHR-dependent fashion. IDO metabolizes tryptophan to kynurenine, an AHR ligand [77,78], reinforcing this loop. Kynurenine, in turn, can also drive Treg formation [79,80,81]. This early work by the groups of Chris Bradfield or Chris Vogel led to further discoveries. These included the AHR-mediated control of DC differentiation and immunosuppressive function [82,83,84], the reduced function of DC in influenza infection [85], the suppression of central nervous system autoimmunity [86], or the use of this pathway for glioblastoma tumor cells to combat their attack by T cells [87].

After years of research, it is fair to say that the immune system is a hotspot for AHR-signaling in the body, regulating both differentiation from stem cells and many specific functions of individual subsets. 

## 4. Ligand Diversity—A Challenging Question Then and Now

**Figure 1:**
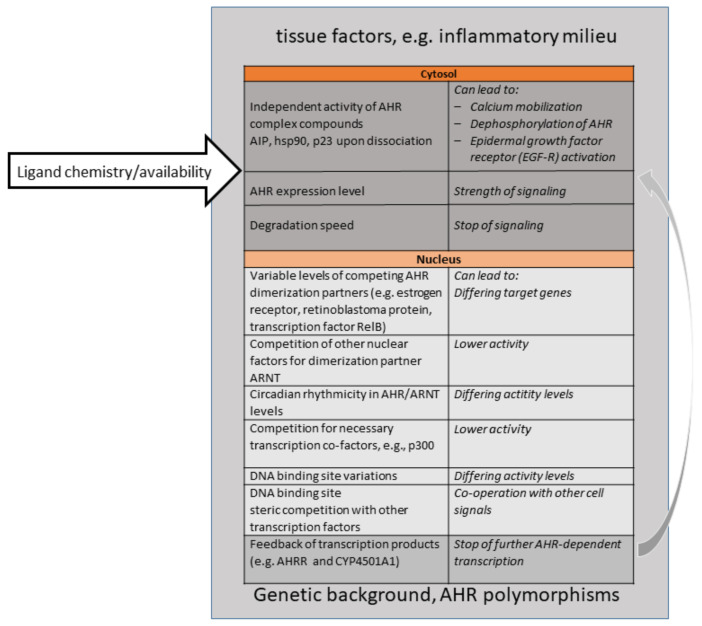
Overview of Major Parameters Influencing AHR signaling outcomes. Please see references in Text and Table 1 for details.

Importantly, this shift towards appreciating the physiological roles of AHR came along with a better knowledge of “natural” ligands, understood as ligands not produced by human industrial activities. AHR binds to a large number of ligands, which only have a few features in common, such as a certain size and planarity [4,88,89]. This “promiscuity” had been known for a long time. Natural ligands can be endogenous, i.e., produced by one’s own body or one´s own gut microbiota, or be ingested (partly as precursors) as part of a plant-based diet. Examples are the amino acid metabolites kynurenines, 2-(1′ H-indole-3′-carbonyl)-thiazole-4-carboxylic acid methyl ester (ITE), or FICZ, and plant-derived glucosinolates, and indoles. Importantly, the endogenous production and dietary uptake of ligands are necessary for sufficient AHR signaling levels; they are thus critical for health. This became obvious early on, when experiments demonstrated that the removal of AHR ligands from the diet in mice resulted in effects similar to genetic AHR deficiency. For instance, an AHR-ligand-low diet led to a sharp drop in the frequency of protective intraepithelial lymphoid cells type3 in the gut, higher susceptibility to gut inflammation and infection severity by the murine gut pathogenic bacterium *Citrobacter rodentium* [90], as well as the loss of γδ T cells (a lineage subset of T cells bearing a γδ T cell receptor) in the gut and in the skin [91,92]. An AHR-ligand-low diet impairs the skin barrier, similar to the effect seen in genetically AHR-deficient mice [93]). Cruciferous plants, (cabbage, kale, broccoli, rape seed etc.) are especially rich in glucosinolates, precursor compounds for AHR ligands [94]. “Eat your veggies”, was thus a popular piece of advice resulting from these mouse experiments on protective AHR-activity in the gut [91,95]. However, be aware; toxicologists know that dose is relevant. When people replace vegetables with high-dose dietary supplements of, e.g., indole-3-carbinol (I3C), they might be at risk, as at a high dosage, I3C can become pro-carcinogenic. For a discussion of this and the discovery of “natural” ligands, see the review by Hubbard et al. from 2015 [96]. To date, it has largely been unclear as to what extent the ligand structure and chemistry (such as non-covalent binding options) govern the observed outcome of AHR activation. Ligands may modulate the conformation changes in liganded AhR-, and thereby the intensity of nuclear translocation, the affinity towards other nuclear heterodimer partner molecules (AHR-Represssor, RelA, Estrogen receptor etc.), the initiation of transcription, or the susceptibility of AHR to degradation. Other inflammatory and hormonal signaling pathways may modulate the outcome of AHR signaling as well, e.g., a cooperation with nuclear factor (NF)κB or the estrogen receptor. In addition, epigenetic factors can influence the overall outcome of AHR-signaling, such as the accessibility of promoters or concomitant availability of other factors in the transcription machinery. Experiments to address these questions would require AHR crystallization or sophisticated biochemistry, both of which have not caught on, and few groups are working on this. Figure 1 gives a visualization of the numerous influences which shape AHR signaling in cells or tissues, and which are relevant for therapeutic targeting of the AHR signaling pathway, especially upon systemic exposure to ligands.

## 5. AHR in the Skin 2000

As stated above, barrier organs, the lung, skin and gut, are particularly rich in AHR protein. In parallel to research about T cells and immunity, which was rapidly increasing from 2008 onwards, barrier organs became interesting as major sites of contact to the environment, and thus to AHR ligands. It seemed natural to look into this as well. With the new century, dermatologist Jean Krutmann, as the new director in my own institute, extended focus to skin research, integrating toxicology, epidemiology and immunodermatology. The link to toxicology was obvious; in humans, the oldest observation of dioxin toxicity is chloracne, which had already been described in the late 19th century. The chloracne-typical acneiform lesions are hamartomas, paralleled by severe atrophy of the sebaceous glands [97]. Epidemiological evidence indicated a link between skin cancer, other skin diseases and AHR in the presence of environmental/occupational HPAH and PAH exposure in workers (such as coal miners) or by lifestyle (e.g., smokers) [98]. Soon, research revealed that all skin cells, not just keratinocytes, express AHR, and sometimes its repressor (AHRR): fibroblasts, Langerhans cells, dermal DC, dendritic epidermal T cells, mast cells, dermal endothelial cells, melanocytes, dorsal root ganglia/sensory neurons, and others [92,99,100,101,102,103]. 

The immunological challenge for the skin is the balance between protecting against a constant threat of pathogens, and suppressing unnecessary and harmful inflammation. High-energy UV radiation from the sun harms the skin. The repair of DNA damage, removal of dead cells if necessary, and surveillance against cancer cells is, therefore, another vital task of the skin [104]. I began with research on Langerhans cells (LC) in the skin. LC are resident dendritic cells, which take up antigen, mature and migrate to the next lymph node. There, they present the antigen to T cells, which gain the ability to move into the skin to the site of antigen exposure. We found that LCs, like other DCs, are rich in AHR. Work with AHR-deficient mice then demonstrated that AHR controls LC maturation and function [103], resulting in an impaired skin immune response. This is just an example. More research at my institute and by other groups, demonstrated, within a few years, that AHR is expressed in and controls typical functions of other skin cell types as well: pigment production and UV-induced proliferation by melanocytes, homeostasis and the inflammatory default setting of dendritic epidermal T cells, dermal fibroblast matrix metalloproteinase 1 production [105,106,107]. Work on keratinocytes, the epidermal structural cells, had been done previously, as stated above [14,108,109]; furthermore, the role of AHR for filaggrin production, and thus the skin barrier, had been discovered in 2011 by Thomas Sutter’s and Masutaka Furue’s groups in the USA and Japan, respectively [110,111,112,113]. In 2007, Ellen Fritsche and Jean Krutmann reported the UV-damage response by keratinocytes [114], and three years ago, together with Thomas Haarmann-Stemmann, demonstrated in AHR-deficient mice that AHR participates in the decision of a UV-damaged cell to repair DNA-damage or go into apoptosis [115]. In summary, we know today that AHR signaling is critical for healthy skin, being involved in: detoxification [116], barrier integrity [93], immunity [92,103], skin homeostasis, the DNA damage response [115,117], pigmentation [101,118]. It is also involved in (environmentally mediated) skin pathogenesis, especially overshooting inflammation like eczema, barrier impairment of the epidermis, UV-damage and induction of Treg cells by UV, or skin cancer. While it is beyond the scope of this review to discuss the large and exciting area of AHR and cancer, it may be noted here that there is a strong link between AHR’s effects on immune cells and on cancer. For instance, through AHR-involvement in the regulation of checkpoint-inhibitors enforcing T-cell anergy, or by AHR-inducing drugs such as verumafenib, which interfere with inflammation [119,120,121,122]. Table 2 gives a list of skin diseases, where AHR signaling is relevant and may be considered as a target for therapy. Because many skin diseases cause severe suffering or distinctly impair the quality of life, novel therapeutic approaches, such as targeting AHR-signaling, are imperative. In line with other research fields, which saw therapeutic options in targeting the AHR (see the report from the 4th International AHR meeting in 2018 [123]), dermatology groups worldwide are looking into this at present. 

### Therapeutic Approaches for Skin Diseases–Ancient and Complex

As stated above, AHR is highly expressed in the skin. While activation might come from “within”, e.g., via the diet, AHR-ligands are also produced locally in and on the skin. Importantly, the UV-generated tryptophan dimer FICZ, a high-affinity AHR-ligand, is ubiquitous in the skin and found systemically [124,125]. Molecules secreted by the common skin-residing fungi *Malassezia spec*., or by certain bacteria, as discovered more recently, provide ligands as well [126,127]. Externally applied cosmetics or lifestyle products might also contain AHR-ligands; some have been in use for centuries. For instance, the naphtoquinone lawsone, a major pigment in henna products, was shown to be an AHR ligand, and can have an ameliorating capacity in atopic dermatitis [128]. Concerning drugs, medicinal coal tar is an ancient treatment for skin inflammation, especially psoriasis and skin disease [129,130,131]. Recent work by Ellen van den Bogaard from the Netherlands showed that the active chemicals in coal tar are its various polycyclic aromatic hydrocarbons. Coal tar restores filaggrin expression and is anti-inflammatory, antagonizing IL-4 and IL-13, as well as inducing Nuclear Factor Erythroid 2-Related Factor 2 (NRF2). Together, this results in the long-known effect of amelioration of the disease [129]. Of note, beyond AHR-binding activity, the efficacy might also involve changes in the skin microbiome, which could also be the case for tapinarof, another effective anti-inflammatory drug and AHR-ligand used against atopic dermatitis and psoriasis [132]. 

It remains a challenge to work out and understand the background skin conditions which turn AHR into what, in 2015, Jean Krutmann, Thomas Haarmann-Stemmann and I called a “Janus-faced” signaling pathway [133]. Diseases and conditions for which treatment options are under consideration are (i) the inflammatory conditions atopic dermatitis (as discussed above) and eczema, and psoriasis on the one side (ii) melanoma and non-melanoma skin cancer, (iii) pigmentation and intrinsic and extrinsic skin ageing (see Table 2). 

Beyond the topical application of AHR ligands, a very new and exciting line of research concerns the gut–skin axis and the role of the diet. It is, of course, well-known that dietary antigens can result in immune reactions of the skin, such as allergic rashes, so such an axis is obvious. Together with Jean Krutmann, my group showed, in 2015, that dietary ligands can reach the skin, and that both lack of AHR and lack of AHR-ligands in the diet impair skin barrier functions [93]. Moreover, feeding with I3C metabolites improved the skin barrier. I3C from dietary plants is a precursor ligand that is converted by the acidic milieu of the stomach to 3,3′diindolylmethane (DIM), 2-(indol-3-ylmethyl)-3,3′-diindolylmethane (LTr-1), and indolo [3,2-*b*]carbazole (ICZ) [134,135]. It is well documented that AHR activity regulates gut bacteria abundance and composition [136]. Both genetic deletion of the AHR or dietary removal of AHR ligands result in a higher bacterial load and dysbiosis [91]. 

A healthy and physiologically critical AHR activity in the gut is driven not only by dietary ligands, but also by gut bacterial metabolites [137,138,139], and thus dysbiosis may affect healthy AHR activity levels. This can affect the generation of Treg cells, and thereby modulate the anti-inflammatory tonus spreading beyond the gut [79,140]. The microbial gut–skin axis is likely to be based on molecules (or their metabolites generated in the liver by first-pass effects) produced by the bacteria. Examples include indole derivatives, retinoic acid (both driving Treg generation), urolithins (an AHR antagonist, [141]) or the short chain fatty acid (SCFA) butyrate with its promotion of regulatory T cells, inhibitory effects on cytokine production and NF-κB signalling [142]. Intriguingly, it was demonstrated that, in both mice and humans, supplementation with various Lactobacillus species improved the skin barrier [143]. This research is still in its infancy, and more work is needed to completely understand this complexity and eventually harness it for prevention and therapy. 

## 6. Conclusions and Open Questions

As the work on the thymus and skin shows, AHR research has come a long way, moving from a molecule of sinister reputation, as the mediator of the toxicity of environmental pollutants, to a transcription factor with ever-increasing and exciting functions in physiology. Currently, despite the amount of data, we do not fully understand the complex conditions, which govern the outcome of AHR activity for a given tissue. While I think that gaining such a complete picture is critical and important, therapies based on AHR modulators (agonists, antagonists or molecules somehow interfering with AHR signaling) are already happening in an incremental, trial-and-error fashion as compounds are being tested. In any case, the trajectory of AHR highlights the benefits research gains from interdisciplinary approaches. An open mind towards findings in other fields than one’s own can be very fruitful for one’s own research.

## Figures and Tables

**Table 1:** Major Milestones of Discovery related to aryl hydrocarbon receptor (AHR) signaling: biochemistry, genetics, and function ^a^.

Year	Discovery	Reference
1976	Aryl hydrocarbon receptor (AHR) identified	[29]
1991	An Ultraviolet (UV)-photoproduct from tryptophan activates “aryl hydrocarbon hydroxylase” (now Cytochrome P (CYP) 450)	[30]
1992	Cloning of the murine AHR	[31]
1992	Discovery of AHR nuclear translocator (ARNT)	[32]
1993	Cloning of the human AHR	[33]
1993	ARNT is the dimerization partner of AHR	[21]
1994	Core sequence of dioxin responsive element (DRE) 5´-GCGTG-3´ identified in CYP4501A1 promoter	[34]
1995	Toxic Equivalency Factors concept	[35]
1995, 1996, 1997	Three AHR-deficient mouse lines independently generated	[36,37,38]
1996	lack of AHR abrogates TCDD toxicity	[39]
1998	Formylindolo[2,3-b]carbazol FICZ, a possible endogenous ligand	[40]
1999	AHR-repressor (AHRR) identified	[41,42]
2000	Generation of an ARNT deficient mouse	[43]
2004	Comparative assessment of DREs in three species	[44]
2007	microarrays show cell-specific gene expression triggered by AHR	[45]
2007	AHR is part of cullin 4B ubiquitin ligase complex, CUL4B(AhR)	[46]
2008	AHR-Repressor deficient mouse generated	[47]
2013	Crystal structure of the AHR PAS-A domain	[48]
2013	First reports concerning AHR and microRNA induction	[49,50]
2014	AHR is a quorum sensor for bacteria	[51]
2016	low AHR affinity to polycyclic aromatic hydrocarbon in humans by evolutionary selection pressure	[52]
2017	Crystal structure of AHR/ARNT dimer and AHRR/ARNT	[53,54]
2017	Forced CYP over-expression depletes AHR ligands, resulting in a quasi AHR-ligand deficient state.	[55]

^a^ this table is an overview, with no claim to completeness.

**Table 2:** Skin diseases with AHR-targeted treatment opportunities.

Disease
Psoriasis
Atopic dermatitis
Pigmentation, Vitiligo
Wound healing
Non-melanoma skin cancer
Melanoma skin cancer
Premature extrinsic skin aging by air pollution
UV-irradiation and DNA damage
Eczema
Hidradenitis suppurativa/Acne inversa
Contact hypersensitivity
Chloracne management

## Abbreviations

AHR	Aryl hydrocarbon receptor;
AHRR	Aryl hydrocarbon receptor repressor
DC	dendritic cell;
FICZ	6-formylindolo[2,3-b]-carbazol;
PAH	polycyclic aromatic hydrocarbons;
HPAH	halogenated polycyclic aromatic hydrocarbons;
IL	interleukin
TCDD	2,3,7,8-tetrachlorodibenzo-p-dioxin;
Treg, Th	regulatory T cell; T helper cell
UV	ultraviolet
